# Optimum design of reference points distribution in three-dimensional reconstruction of dental model in intercuspal position

**DOI:** 10.1186/s12903-021-01919-z

**Published:** 2021-11-03

**Authors:** Yujia Wu, Zhewen Hu, Xinyue Zhang, Hefei Bai, Yuchun Sun, Baolin Fan

**Affiliations:** 1 Center of Digital Dentistry, Peking University School and Hospital of Stomatology, 22 Zhongguancun Ave South, Haidian, Beijing, 100081 People’s Republic of China; 2 Department of VIP Dental Service, Peking University School and Hospital of Stomatology, 22 Zhongguancun Ave South, Haidian, Beijing, 100081 People’s Republic of China; 3 Department of Prosthodontics, Peking University School and Hospital of Stomatology, 22 Zhongguancun Ave South, Haidian, Beijing, 100081 People’s Republic of China; 4 Department of Medical Equipment Management Division, Peking University School and Hospital of Stomatology, 22 Zhongguancun Ave South, Haidian, Beijing, 100081 People’s Republic of China; 5 National Engineering Laboratory for Digital and Material Technology of Stomatology, National Clinical Research Center for Oral Diseases, Beijing Key Laboratory of Digital Stomatology, Research Center of Engineering and Technology for Digital Dentistry of Ministry of Health, Beijing, People’s Republic of China

**Keywords:** 3D scanning, Dental models, Digital technology, Imaging, Three-dimensional, Intercuspal relation, Technology, Dental

## Abstract

**Purpose:**

The scanning of plaster models for three-dimensional (3D) construction requires their rigid fixation in the intercuspal position. Factors such as installation, motion, and scanning procedures influenced the accuracy of this method, which ultimately influence the results. Therefore, the present study attempted to provide an optimal and accurate method with less complex procedures and a more accessible equipment for determining the intercuspal relation in the 3D occlusal construction of dental models.

**Methods:**

A pair of plastic mounting plates that could be directly attached to a mechanical articulator was designed and 3D printed. Nine axial hemispherical concaves were introduced on the axial surface of each plate. The rigidly fixed maxillary and mandibular dental models were scanned directly. The distances *D*_*R*_ between nine pairs of concaves on both mounting plates adhered to the maxillary and mandibular sections of the articulator were measured using the three-coordinate measuring machine Faro Edge as the reference. The present study comprised seven test groups varying in number and location. Assessing the reference points from each of the seven groups performed the 3D construction. The Geomagic Studio software was used to construct the concaves of digital casts, and the distances *D*_*M*_ between the pairs of concaves were measured as test values. Variable differences between *D*_*R*_ and *D*_*M*_ were analyzed.

**Results:**

An optimum distribution scheme was obtained for reference point registration by quantitatively evaluating accuracy levels of the 3D constructions of different reference point distribution patterns. This scheme can serve as a reference for related studies and dental clinic operations.

**Conclusions:**

Three-dimensional construction of the intercuspal relation during scanning of the maxillary and mandibular models with an accuracy of 0.046 mm ± 0.009 mm can be achieved using the improved design of mounting plates.

## Background

Digital dentistry has advanced remarkably in the past 50 years. Digital dental technology originated in 1971 when the first dental computer-aided design and computer-aided manufacturing (CAD/CAM) prototype system Sopha was developed by Professor Duret [1–5]. Application of the digital technology in dentistry has changed the entire dentistry workflow, influencing processes such as diagnosis, design of dental restorations, planning and execution of treatment procedures, and exchange and storage of patient data [6].

The analogue data can be transferred into virtual dental space through direct and indirect scanning workflows [7, 8]. The indirect digital workflow involves a physical impression and scanning of plaster models [9]. Plaster models are poured from conventional impressions, and the maxillary and mandibular models are then separately scanned using a desktop optical scanner. These models are mounted in the intercuspal position (ICP) with an interocclusal record and digitalized. The CAD system requires the three-dimensional (3D) construction of the ICP relation between dental models, which is achieved by scanning maxillary, mandibular, and ICP relation models using a dental model 3D scanning system. This process requires either the insertion of the physical articulator with paired maxillary and mandibular models into the desktop optical scanner or the transfer of the paired maxillary and mandibular models through the physical articulator (e.g., inEos X5; Dentsply Sirona, York, PA, and Smart Optics 880 Dental Scanner; Smart Optics, Bochum, Germany) or through a transfer kit (e.g., Ceramill Map400; Amann Girrbach, Koblach, Austria and Smart Optics Vinyl; Smart Optics, Bochum, Germany), a plate (e.g., 3Shape D2000; 3Shape, Copenhagen, Denmark) or a patented assisted device (e.g., i3Dscan; Imetric 3D SA, Courgenay, Switzerland) [10–12].

This complicated process requires a pair of plaster models to be rigidly fixed at the ICP and presents the following challenges:

- Models must be installed on an articulator, a transfer kit, or a plate for a third scanning [13–15]. Thus, errors may be introduced when the ICP relation models are taken down from the articulator, broken off, and refastened in the scanner cavity.
- The paired plaster model is more than twice the volume and weight of a single model, requiring a larger capacity and scanning area for the scanner.
- A scanner basically consists of a light source, one or more cameras, and a motion system supporting several axes for positioning the scanned object toward the light source and cameras [4]. The motion system tilts, rotates, and translates the object during scanning, which may easily result in a slight relative displacement between the paired models, thereby introducing errors into the ICP.

The mounting plate was connected to both maxillary and mandibular models and the articulator via magnets [16, 17]. The present study attempted to demonstrate the utility of an improved pair of mounting plates that can be mounted onto a mechanical articulator for the 3D construction of the ICP relation of a dental model. Nine hemispherical concaves were introduced on the axial surface of the mounting plate, and the centre points of these concaves at different positions were selected for the 3D construction of the ICP relation between the maxillary and mandibular models. The present study also quantitatively evaluated the accuracy of 3D constructions using different reference point distributions, thereby obtaining an optimal distribution scheme for reference point registration to serve as a reference for related studies and dental clinic operations.

## Methods

### Design and manufacture of the mounting plate

**Fig. 1:**
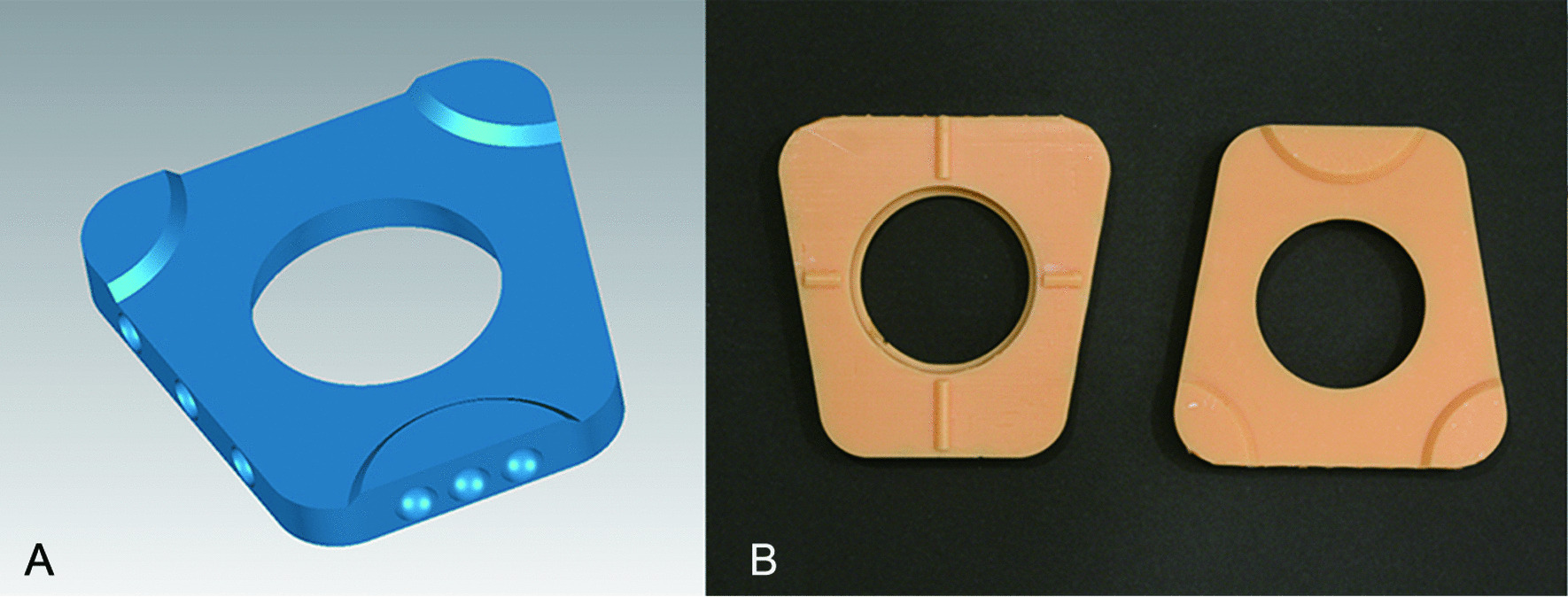
**A** Mounting plate with hemispherical concave. **B** Mounting plates after 3D printing

The 3D data describing the mounting plate of an articulator (Amann Girrbach, Koblach, Austria) was obtained using a 3D dental model scanner (Activity 880, Smart Optics, Bochum, Germany). Three 6-mm-diameter hemispherical concaves were designed using SolidWorks 2015 (Dassault Systemes S.A, Paris, France) and introduced on the mounting plate’s front, left, and right surfaces. A high-precision 3D print was produced (EnvisionTEC Perfactory DD, EnvisionTEC, Gladbeck, Germany). The hemispherical concaves on the right, left, and front-lateral surfaces of the maxillary and mandibular mounting plates were named according to the Federation Dentaire International specifications. The central hemispherical concave on the front surface of the maxillary and mandibular mounting plate was named 0 and 0′ respectively (Fig. 1). The centre points of the hemispherical concaves were used as the reference points for the 3D construction of maxillary and mandibular models. Additionally, each centre point of the hemispherical concave corresponding to the maxillary mounting plate was paired with the mandibular mounting plate. Nine centre-point pairs were defined, namely 11–41, 12–42, 13–43, 14–44, 0–0′, 21–31, 22–32, 23–33, and 24–34.

### Establishment of the control group and an observation coordinate

**Fig. 2:**
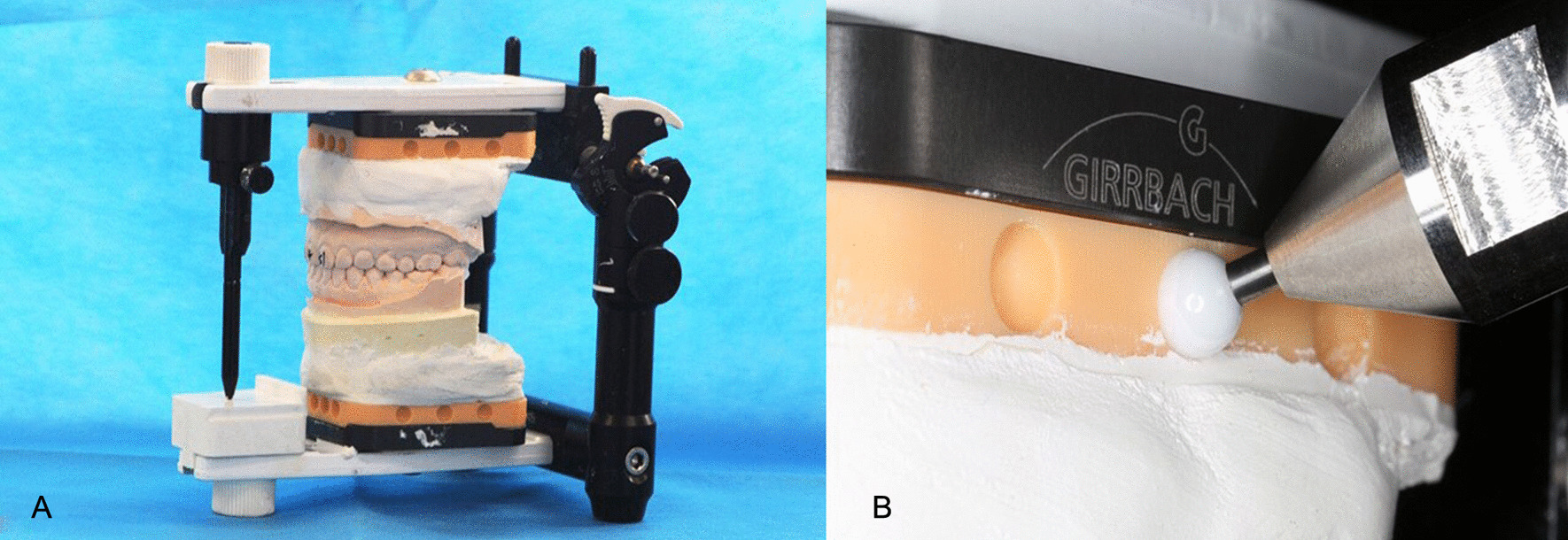
**A** Mounting model onto articulator using the proposed mounting plates. **B** Centre-point measurement using the contact method

The mounting plates with hemispherical concaves were fixed onto the articulator, and the scale interval of the incisal guidance pin was adjusted to zero. A pair of dental models were cast using die stone and mounted at the ICP onto the articulator. A 7-axis Faro Edge contact measurement system (Faro Technologies, Lake Mary, FL, USA) mechanical 3D measurement arm with a contact measurement accuracy of 0.024 mm and a scanning accuracy of 0.059 mm was used to measure the centre-point coordinates of the hemispherical concaves in the maxillary and mandibular sections of the articulator, and the spatial jaw relation of the models in occlusion was obtained (Fig. 2). This step was repeated thrice, and the mean centre-point coordinates obtained from the three measurements were considered the standard data and saved in the STL format. These standard data were then imported into Geomagic Studio 2013. A plane was determined based on three points, namely 12, 0, and 22, and was referred to as the XOY plane. Point 0 was considered as the coordinate origin. The X-axis was considered parallel to the line between Points 12 and 22, and an “observation coordinate system” was established based on Descartes’ rule of signs. The X-axis of the coordinate system was horizontal from left to right, the Y-axis ran horizontally in the anterior–posterior direction, and the Z-axis was vertical. Measured data were termed standard data and saved in the WRP format, a particular file format for software of the Geomagic series.

### Establishment of the test group

A 3D dental model scanner was used to obtain 3D data of the maxillary and mandibular models with the mounting plates. The measured data was imported into Geomagic Studio 2013, and the best fit spherical feature was extracted from point data of each hemispherical concave and its centre’s coordinates were recorded. This step was also repeated three times, and the mean coordinates obtained during the three measurements were calculated and named the fitting data. The maxillary and mandibular models with corresponding fitting centre points of the hemispherical concaves were named the maxillary and mandibular data sets and saved in the WRP format.

### Grouping and 3D construction

**Fig. 3:**
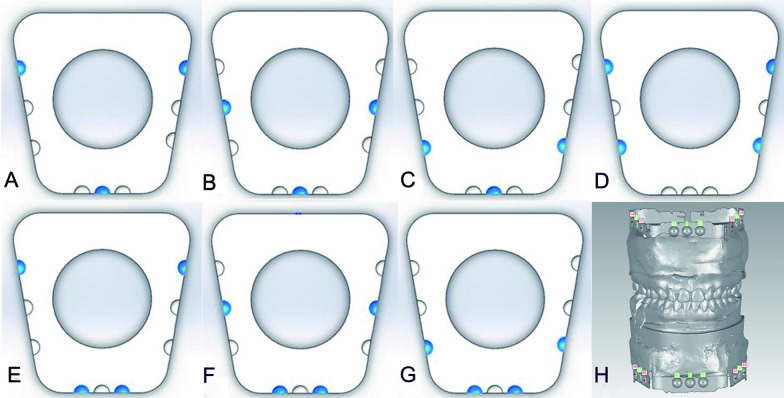
Location of reference pairs: **A** Group 1 (reference pairs 14–44, 0–0′, and 24–34). **B** Group 2 (reference pairs 13–43, 0–0′, and 23–33). **C** Group 3 (reference pairs 12–42, 0–0′, and 22–32). **D** Group 4 (reference pairs 14–44, 12–42, 22–32, and 24–34). **E** Group 5 (reference pairs 14–44, 11–41, 21–31, and 24–34). **F** Group 6 (reference pairs 13–43, 11–41, 21–31, and 23–33). **G** Group 7 (reference pairs 12–42, 11–41, 21–31, and 22–32). **H** The 3D data of the maxillary and mandibular models with the mounting plates

The maxillary, mandibular, and standard data sets were imported into Geomagic Studio 2013. The test group was divided into the following seven subgroups according to the number and distribution of the reference points (Fig. 3):

1. Pairs 14–44, 0–0′, and 24–34 were used as the reference points in group 1;
2. Pairs 13–43, 0–0′, and 23–33 were used as the reference points in group 2;
3. Pairs 12–42, 0–0′, and 22–32 were used as the reference points in group 3;
4. Pairs 14–44, 12–42, 22–32, and 24–34 were used as the reference points in group 4;
5. Pairs 14–44, 11–41, 21–31, and 24–34 were used as the reference points in group 5;
6. Pairs 13–43, 11–41, 21–31, and 23–33 were used as the reference points in group 6; and
7. Pairs 12–42, 11–41, 21–31, and 22–32 were used as the reference points in group 7.

Fitting data in the maxillary and mandibular data sets were aligned to the standard data set by using the reference points of these seven groups to complete the 3D construction of the ICP relation between the maxillary and mandibular models.

### Data measurement

The distance *D*_*R*_ between the paired centre points of the hemispherical concaves in the mounting plates was obtained by the mechanical coordinate measuring system Faro Edge as a reference value. The Faro Edge was calibrated according to the manufacturer’s recommendations. The distance *D*_*M*_ between the paired centre points not used for alignment was measured using the Geomagic Studio 2013 package. The alignment and distance measurement processes were performed fifteen times by each of two operators (operator 1-Wu YJ, operator 2-Hu ZW) in all groups (n = 15 for each of the seven groups by each operator, total N = 210 for two operators). The variable differences between *D*_*R*_ and *D*_*M*_ were calculated. The average of each group’s root mean square error (RMSE) value was considered the representative. The accuracy has defined a combination of trueness (closeness of measured values during repeated measurements) and precision (closeness of measured values during repeated measurements) by the International Standards Organization (ISO 5725-1:1998). The RMSE value was used to quantify the trueness and precision. The intraclass correlation coefficient (ICC) was calculated to evaluate the correlation between the two operators.

### Statistical analysis

The statistical analysis of the data was performed using IBM SPSS Statistics Version 20.0. The Shapiro–Wilk test found normality in the data distribution, and according to Levene’s test, the homogeneity of variance was satisfied. The averages of RMSE values of the differences between paired points from the test and control groups were calculated. For trueness and precision, the averages of the RMSE values of seven groups were analysed using the one-way analysis of variance (ANOVA) with Turkey’s multiple comparisons test. The ICC estimates and their 95% confidence intervals were calculated using SPSS package based on single-measurement, absolute-agreement, 2-way mixed-effects model for intra-operator reliability and single-measurement, consistency, 2-way random-effects model with two operators [18]. The level of significance was set at 5% for all comparisons (*p* < 0.05).

## Results

The RMSE values of trueness and precision of the distance between paired centre points not used for alignment are enumerated in Table 1, which provide the accuracy of the 3D construction. The RMSE value of trueness was the lowest using pairs 14–44, 12–42, 22–32, and 24–34 from group 4 as reference pairs for 3D construction (0.046 mm ± 0.009 mm), which was statistically significant compared with other six groups (*p* < 0.001, Table 2). The RMSE values for trueness were the highest in group 7, which used pairs 12–42, 11–41, 21–31, and 22–32 as reference pairs for 3D construction (0.124 mm ± 0.016 mm). Group 7and 3 showed significantly lower trueness than the other six groups (*p* < 0.05, Table 2). The difference between the performances of these two groups was statistically significant.

**Table 1:** Results of the measurements of the root mean square error (RMSE) values of trueness and precision

Group	Trueness (Mean ± SD) (mm)	Precision (Mean ± SD) (mm)
1	0.076 ± 0.010	0.019 ± 0.009
2	0.080 ± 0.009	0.021 ± 0.007
3	0.107 ± 0.011	0.028 ± 0.011
4	0.046 ± 0.009	0.022 ± 0.009
5	0.072 ± 0.007	0.017 ± 0.007
6	0.079 ± 0.008	0.018 ± 0.008
7	0.124 ± 0.016	0.027 ± 0.011

SD, standard deviation

**Table 2:** Mean ± standard deviation and *p*-values between each group’s RMSE values of trueness

	Mean ± SD	Group 1	Group 2	Group 3	Group 4	Group 5	Group 6	Group 7
Group 1	0.076 ± 0.010	N/A	0.123	< 0.001*	< 0.001*	0.272	0.999	< 0.001*
Group 2	0.080 ± 0.009	0.123	N/A	< 0.001*	< 0.001*	< 0.001*	0.037	< 0.001*
Group 3	0.107 ± 0.011	< 0.001*	< 0.001*	N/A	< 0.001*	< 0.001*	< 0.001*	0.007*
Group 4	0.046 ± 0.009	< 0.001*	< 0.001*	< 0.001*	N/A	< 0.001*	< 0.001*	< 0.001*
Group 5	0.072 ± 0.007	0.272	< 0.001*	< 0.001*	< 0.001*	N/A	0.547	< 0.001*
Group 6	0.079 ± 0.008	0.999	0.037	< 0.001*	< 0.001*	0.547	N/A	< 0.001*
Group 7	0.124 ± 0.016	< 0.001*	< 0.001*	0.007*	< 0.001*	< 0.001*	< 0.001*	N/A

SD, standard deviation

N/A = “Not applicable”; * *p* < 0.05

Among the groups containing three points, the highest trueness was achieved using pairs 14–44, 0–0′, and 24–34 from group 1 as reference pairs, whereas the lowest trueness was achieved using pairs 12–42, 0–0′, and 22–32 from group 3 as reference pairs. Group 1 demonstrated the most homogenous distribution of reference pairs among the three reference pair groups, whereas group 4 demonstrated the most homogenous distribution among the four reference pair groups. The RMSE value of precision was highest for group 3 (0.028 ± 0.011), followed by group 7 (0.027 ± 0.011) and group 4 (0.022 ± 0.009) (Table 3). There was no significant difference between the RMSE values for the precision of groups 3, 4 and 7 (*P* > 0.05, Table 3).

**Table 3:** Mean ± standard deviation and *p*-values between each group’s RMSE values of precision

	Mean ± SD	Group 1	Group 2	Group 3	Group 4	Group 5	Group 6	Group 7
Group 1	0.019 ± 0.009	N/A	0.979	0.001*	0.831	0.995	1	0.006*
Group 2	0.021 ± 0.007	0.979	N/A	0.018*	0.999	0.752	0.872	0.082
Group 3	0.028 ± 0.011	0.001*	0.018*	N/A	0.075	< 0.001*	< 0.001*	0.998
Group 4	0.022 ± 0.009	0.831	0.999	0.075	N/A	0.43	0.586	0.249
Group 5	0.017 ± 0.007	0.995	0.752	< 0.001*	0.43	N/A	1	0.001*
Group 6	0.018 ± 0.008	1	0.872	< 0.001*	0.586	1	N/A	0.001*
Group 7	0.027 ± 0.011	0.006*	0.082	0.998	0.249	0.001*	0.001*	N/A

SD, standard deviation; N/A = “Not applicable”; * *p* < 0.05

In order to determine the intra- and inter-operator reliability, the ICC was calculated. Intra-operator ICC showed an excellent reliability (ICC > 0.9) for 2 operators (Table 4). Excellent inter-operator reliability (ICC > 0.9) was observed for group 1, 2, 3 and group 7, while good inter-operator reliability (ICC > 0.75) was observed for group 4, group 5 and group 6 (Table 5).

**Table 4:** Intraclass correlation coefficient (ICC) estimates for intra-operator reliability and their 95% confidence intervals

	Operator 1		Operator 2	
Group	ICC	95% Confidence interval (Lower bound–upper bound)	ICC	95% Confidence interval (Lower bound–upper bound)
1	0.984	0.956–0.997	0.976	0.937–0.996
2	0.992	0.975–0.999	0.982	0.953–0.997
3	0.984	0.956–0.997	0.985	0.961–0.998
4	0.987	0.958–0.999	0.979	0.941–0.997
5	0.983	0.951–0.998	0.966	0.906–0.996
6	0.990	0.968–0.999	0.978	0.937–0.997
7	0.981	0.945–0.998	0.986	0.961–0.998

**Table 5:** ICC estimates for inter-operator reliability and their 95% confidence intervals

Group	ICC	95% Confidence interval (Lower bound–upper bound)
1	0.938	0.827–0.979
2	0.936	0.822–0.978
3	0.949	0.856–0.983
4	0.924	0.788–0.974
5	0.877	0.672–0.957
6	0.915	0.766–0.971
7	0.947	0.850–0.982

## Discussion

Sun et al. [19] and Yuan et al. [20] have indicated that the errors in jaw relationship construction can be controlled to values within approximately 100 μm based on the model spatial relationship localization device and the common region registration method supported by the dental model 3D scanner. The iterative closest point algorithm was used for the registration process. This algorithm iterates the rigid transformation between two models to minimize the alignment error and registers the spatial geometric relationship between models. This closest point registration technique iteratively solves for the nearest corresponding point, establishes a transformation matrix, performs repeated transformations on one of the two models until convergence is achieved and then stops. Mass points are employed during the iterative closest point registration. A relatively noticeable change is required in the curvature of the model surface because the iterative closest point registration process demonstrates poor accuracy when employed on models with a low curvature change.

The Reference Point System (RPS) alignment method refers to the movement of one or more objects to share a coordinate system position based on three or more paired reference points. When registering with the RPS method, selecting reference point pairs and weight settings may affect the final registration results. Li et al. [21] added registration markers to an edentulous jaw model to complete 3D construction when only the maxillary and mandibular jaw models were scanned. Their proposed method eliminated the subjective error incurred during the selection of feature points and prevented the displacement and rotation of the paired model at the central relation. However, markers for each model still must be prepared, and the complex operation of locating centre points must be performed using the contact measurement system. Hu et al. [22] developed a 3D construction method by scanning maxillary and mandibular jaw models through a mechanical appliance with markers. The markers were not required to prepare and measure the centre points for each model. However, the inability of the appliance to be docked with a conventional articulator made its use difficult in clinics. In the current study, we went beyond the previous work by independently developing a pair of mounting plates with hemispherical concaves that can fit with a conventional articulator, facilitating the 3D construction of the dental model jaw relation. All reference points considered in this study were centre points calculated through software and were based on the surface of the hemispherical concaves. Additionally, similar weights were set for each reference point used for alignment to ensure the equal contribution of each reference point to the alignment, reducing the local deviations during 3D construction.

The uniform distribution of hemispherical concaves on the mounting plate represents the spatial positional relationship of the maxillary and mandibular models. The accuracy of the 3D construction of the dental model at the ICP by using the mounting plate with the hemispherical concaves was quantitatively evaluated by measuring the RMSE values of the difference in the straight-line distance between those paired centre points that were not used as the reference points between the test and control groups. Both the number and distribution of different reference points affected the accuracy of the 3D construction.

The reference points of groups 3 and 7 were focused on the front part of the mounting plate, indicating that the reference point location affects the construction accuracy. Three reference point pairs were used for 3D construction in the maxillary and mandibular models of groups 1–3. The reference point distribution of the three pairs was the widest in group 1, whereas it was the most concentrated in group 3. The 3D construction accuracy of group 1 was the highest of the three groups, followed by group 2 and group 3. This finding demonstrates that given the same number of reference point pairs, the 3D construction accuracy is a function of point distribution and that the accuracy increases with the homogeneity of that distribution. Group 1 demonstrated the most homogenous distribution of reference pairs among the three reference pair groups, whereas group 4 demonstrated the most homogenous distribution among the four reference pair groups. The 3D construction accuracy of group 4 was higher than that of group 1, and this difference was statistically significant, suggesting that the accuracy of 3D construction may be related to the number of reference pairs.

The reference pair positions of group 4 are comparable to the distal end of the canine and second molar, indicating the current clinical 3D construction practices should focus on this region. A selection error was observed when the scale interval of the incisal guidance pin was adjusted to 0. Future studies could eliminate those errors. Additionally, materials and manufacturing processes for mounting plates can be improved in future research. Mounting plates can be fabricated with numerically controlled cutting techniques to achieve high accuracy, and metals could be used instead of low-strength plastic as the material for the fabrication of stable and reusable mounting plates. Since the occlusion surface had a distance from the reference pairs, the accuracy of 3D construction can only be taken as indirect evidence. The method of establishing the ICP relation of dental models proposed in this paper can be further improved, enabling a simple yet accurate modeling method.

## Conclusion

In this study, we design a hemispherical concaves surface on the mounting plates to precisely set the spatial position relationship of the reference points. According to the method, the hemispherical concaves can be designed on various brands of mounting plates docked with appropriate articulators. Assuming that the mounting plates and the maxillary and mandibular models are in a rigid body, 3D construction of the dental model ICP relation within an accuracy of 0.046 mm ± 0.009 mm can be achieved using the improved design of mounting plates by only scanning the maxillary and mandibular models. This method greatly simplifies the construction process and achieves the construction accuracy within 50 μm, satisfying the clinical requirement.

## Data Availability

The datasets acquired during and/or analyzed during the current study are available from the corresponding author on reasonable request.
